# Editorial: Pharmacokinetics of herbal medicines and herb-drug interactions

**DOI:** 10.3389/fphar.2022.1107777

**Published:** 2022-12-13

**Authors:** Wei-Wei Jia, Chuang Lu, Ge Lin, Guang-Bo Ge

**Affiliations:** ^1^ State Key Laboratory of Drug Research, Shanghai Institute of Materia Medica, Chinese Academy of Sciences, Shanghai, China; ^2^ Accent Therapeutics, Inc., Lexington, MA, United States; ^3^ School of Biomedical Sciences, Faculty of Medicine, The Chinese University of Hong Kong, Hong Kong SAR, China; ^4^ Shanghai Frontiers Science Center for Chinese Medicine Chemical Biology, Institute of Interdisciplinary Integrative Medicine Research, Shanghai University of Traditional Chinese Medicine, Shanghai, China

**Keywords:** herbal medicine, pharmacokinetics, drug combination, drug interaction, molecular mechanism, drug transporter, drug-metabolizing enzyme

Herbal medicines have provided a basis for health management and disease prevention in many Asia countries, including China, India, and Japan. Several herbal medicines have been evidenced scientifically for their efficacy and safety in rigorous clinical trials (Wang et al., 2011; Li et al., 2013; Shang et al., 2013; Song et al., 2019). In the past few decades, therapeutic regimen combining synthetic drugs with herbal medicines represent a promising strategy for preventing and treating multifactorial diseases. Such combination probably exhibits different mechanisms of actions and improves the pharmacodynamic effects and safeties. The thorough knowledge of mechanisms of and chemical basis for herbal medicine actions on therapeutic targets may provide a better rationale for the understanding of combination of drugs with different mechanisms, which are preferable in the polytherapy of diseases. To support the drug combination, the impact of such combination on therapeutic outcome needs to be assessed, including pharmacokinetic interactions mediated by drug-metabolizing enzymes and drug transporters. Commonly, most of clinically used herbal medicines are prepared from multiple herbs that contain hundreds of chemically diverse constituents, which may interact with a variety of drug-metabolizing enzymes and drug transporters in extremely complex ways (Ge, 2019). The complex interactions make a great challenge for an investigator to quickly and accurately find the key compounds responsible for therapeutic actions of the herbal medicines. Pharmacokinetic research provides a practical approach to identifying potentially important compounds (bioavailable at the action loci with significant exposure levels after dosing an herbal medicine). Better understanding of the pharmacokinetic behaviors of the bioavailable compounds is vital in evaluating therapeutic and adverse effects of the herbal medicine, as well as their pharmacokinetic interactions. This is of particular importance in cases where herbal medicine is added to conventional therapy.

Our Research Topic compiles 11 innovative original research contributions from prominent scientists in the field. This compilation covers a number of herbal medicines in pharmacokinetic-pharmacodynamic (PK-PD) relationships, herb-drug interactions, and the associated molecular mechanisms related to drug-metabolizing enzymes and drug transporters.

Multi-compound pharmacokinetic approach was used to identify potentially important compounds from single herb or herbal combinations and also characterize their pharmacokinetics and disposition (Lan et al., 2021; Li et al., 2022). Based on comprehensive and validated UPLC-MS/MS-bioanalytical assays, the study by Cheng et al. found that among ten main stilbene glycosides and anthraquinones, 2,3,5,4′-teterahydroxystilbene-2-*O*-β-D-glucoside, emodin, citreorosein, and emodin-8-*O*-β-D-glucoside exhibited higher levels of systemic exposure in rats orally dosed with an extract of the roots of *Polygonum multiflorum* Thunb. (Ploygoni multiglori radix in pharmacopeia of China). Besides these compounds, physcion-8-*O*-β-D-glucoside also significantly exposured to the liver and kidneys of rats. Chen et al. investigated that delicaflavone, robustaflavone, 2″,3″-dihydro-3′,3‴-biapigenin, 3′,3‴-binaringenin, and amentoflavone exhibited the highest levels of lung exposure in rats after receiving a flavonoid extract from the whole plants of *Selaginella doederleinii* Hieron, which is used to treat cancers. These five flavonoids were extensively cleared by fecal excretion. Huang et al. compared cellular pharmacokinetics of luteolin, chlorogenic acid, cryptochlorogenic acid, 3,4-dicaffeoylquinic acid, and 4,5-dicaffeoylquinic acid of the whole plants or roots of *Inula cappa* (Buch.-Ham. ex D. Don) DC. in normal RAW264.7 cells and the LPS-induced cells. WuJi pill, an herbal combination, is used for treating irritable bowel syndrome (IBS). Gong et al. reported the pharmacokinetic differences of five representative compounds (berberine, palmatine, evodiamine, rutaecarpine, and paeoniflorin) in normal rats and IBS-model rats after dosing the pill. The molecular mechanism underlying the pharmacokinetic differences was reported that the decreased expression of tight junction proteins resulted in the increased permeability of colon mucosa in the model rats. Another herbal combination TanReQing injection is used to treat respiratory diseases *via* off-label nebulization in China. Huang et al. demonstrated that lung exposure levels of baicalin and oroxyloside after aerosol inhalation were significantly higher than those after i.v. injection. The inhalation of TanReQing with lower dose could achieve the same effect against LPS-induced lung inflammation in mice. The investigation by Zhang et al. successfully established PK-PD correlation of Yangyin Tongnao granules in rats with cerebral ischemia-reperfusion injury.

Herb-drug interactions are widely recognized as an issue for many drug combinations. Zhang et al. efficiently identified that styrax (the medicinal balsam from the trunks of *Liquidambar orientalis* Mill.) exhibited the most potent inhibitory effects on human CYP3A4, following screening over 100 herbal medicines by a fluorescence-based high-throughput assay. Oral administration of this herbal medicine could elevate systemic exposure levels of midazolam and felodipine *via* oral, but not intravenous, administration of the CYP3A-substrate drugs to rats. Further investigations elucidated that seven styrax-derived pentacyclic triterpenoid acids with high inhibition potency toward CYP3A showed high intestinal exposure levels, while their systemic and hepatic exposure levels were extremely low. Kapelemera et al. found that Xiang-Sha-Liu-Jun-Zi Tang (XSLJZT) could significantly increase levels of systemic exposure to paclitaxel in rats when XSLJZT was pretreated for 3, 5, and 7 days. The results of mechanistic study indicated that XSLJZT could inhibit expression of CYP3A4 in Hep-G2 cells (Cyp3a1/2 in rat liver) and CYP3A4 enzymatic activity of human liver microsomes. Drug interaction was also found to have beneficial effects. For instance, liquorice (Glycyrrhizae radix et rhizome), the roots of *Glycyrrhiza uralensis* Fisch., *Glycyrrhiza glabra* L. or *Glycyrrhiza inflate* Bat., a hepato-protective herbal medicine, is used concurrently with pyrrolizidine alkaloid-containing herbs in many traditional Chinese medicine formulas, and no pyrrolizidine alkaloid poisoning cases have been reported with such combination. Wang et al. comprehensively demonstrated that both liquorice aqueous extract and 18β-glycyrrhetinic acid (the primary bioactive constituent of liquorice) exhibited significant hepato-protective effects against retrorsine (a representative pyrrolizidine alkaloid)-induced hepatotoxicity. The molecular mechanism was that 18β-glycyrrhetinic acid mainly and competitively inhibited the formation of metabolic activation-derived pyrrole-glutathione conjugate mediated by rat CYPs, especially Cyp3a1 (an ortholog of human CYP3A4).

Exploring molecular mechanism is critical for elucidation of pharmacokinetics of herbal medicines and drug interactions. Hepatic and/or renal uptake of phenolic acids, originating from the roots of *Salvia miltiorrhiza* Bge. (Salviae miltiorrhizae radix et rhizome) was the crucial steps in their systemic elimination. Lu et al. comprehensively investigated that several human uptake transporters were found to mediate hepatic and/or renal uptake of phenolic acids in a compound-molecular-mass-related manner. Lithospermic acid and salvianolic acid B (both >500 Da) underwent systemic elimination, initiated by OATP1B1/OATP1B3-mediated hepatic uptake. Rosmarinic acid and salvianolic acids D (350–450 Da) underwent systemic elimination, initiated by OATP1B1/OATP1B3/OAT2-mediated hepatic uptake and by OAT1/OAT2-mediated renal uptake. Protocatechuic acid and tanshinol (both <200 Da) underwent systemic elimination, initiated by OAT1/OAT2-mediated renal uptake and OAT2-mediated hepatic uptake. Pang et al. utilized an ultra-sensitive and easy-to-use assay to assess the inhibitory activities of over 100 herbal products against UGT1A1 in living systems, and found that an extract from the leaves of *Ginkgo biloba* L. (Ginkgo folium) and its biflavones (bilobetin, isoginkgetin, sciadopitysin, and ginkgetin) exhibited the most inhibition potency toward UGT1A1.

We believe that the investigators of these articles have made great efforts to gain new insights into the key herbal constituents responsible for herbal medicine’s therapeutic effects, adverse effects, and herb-drug interactions. We are confident that these innovative and important studies will be very useful for the researchers and clinical pharmacologists to better understand the complex interactions between herbal medicines and the human body, which will be very helpful for accurate medication of herbal medicines in clinical settings. Although great advances have been achieved in methodology, techniques, and applications of pharmacokinetic studies, complex chemical composition, chemical basis, and PK-PD correlation for most herbal medicines remain challenges in bringing herbal medicine from empirical medicine into evidence-based medicine and meeting regulatory requirements. With the advancement of *in vitro* and *in vivo* methodology and computational hardware and software, artificial intelligence represents a promising means to predict the pharmacokinetic properties of numerous constituents for an herbal medicine in a more high-throughput and cost-effective manner.
